# Picobirnaviruses: prevalence, genetic diversity, detection methods

**DOI:** 10.18699/VJ20.660

**Published:** 2020-10

**Authors:** A.Yu. Kashnikov, N.V. Epifanova, N.A. Novikova

**Affiliations:** I.N. Blokhina Nizhny Novgorod Research Institute of Epidemiology and Microbiology, Nizhny Novgorod, Russia; I.N. Blokhina Nizhny Novgorod Research Institute of Epidemiology and Microbiology, Nizhny Novgorod, Russia; I.N. Blokhina Nizhny Novgorod Research Institute of Epidemiology and Microbiology, Nizhny Novgorod, Russia

**Keywords:** picobirnavirus, genomic segment, specific genomic fragment, RT-PCR, primer, amplicon, sequencing, пикобирнавирус, сегмент генома, фрагмент сегмента генома, ОТ-ПЦР, праймер, ампликон, секвенирование

## Abstract

This article presents a general overview of the prevalence, genetic diversity and detection methods of picobirnaviruses (PBVs), which are small, non-enveloped icosahedral viruses with a segmented double-stranded RNA genome consisting of two segments taxonomically related to the genus Picobirnavirus of the family Picobirnaviridae. This review of scientific papers published in 1988–2019 provides data on the PBV distribution in the nature and a broad host range. PBV infection is characterized as opportunistic, the lack of understanding of the etiological role of PBVs in diarrhea is emphasized, since these viruses are detected both in symptomatic and asymptomatic cases. The concept of PBV infection as a chronic disease caused by a long-lasting persistence of the virus in the host is considered. Such factors as stress syndrome, physiological conditions, immune status and host age at the time of primary PBV infection influence the virus detection rate in humans and animals. The possible zoonotic nature of human PBV infection is noted due to the capacity for interspecies PBV transmission acquired during evolution as a result of the reassortment of the genome segments of different viruses infecting the same host. Data providing evidence that PBVs belong to eukaryotes and a challenging hypothesis stating that PBVs are bacterial viruses are presented. The need to intensify work on PBV detection because of their wide distribution, despite the complexity due to the lack of the cultivation system, is emphasized. Two strategies of RT-PCR as main PBV detection methods are considered. The genomes of individual representatives of the genus isolated from different hosts are characterized. Emphasis is placed on the feasibility of developing primers with broader specificity for expanding the range of identifiable representatives of the genus PBV due to a huge variety of their genotypes. The importance of effective monitoring of PBV prevalence for studying the zoonotic and anthroponotic potential using metagenomic analysis is highlighted, and so is the possibility of using PBV as a marker for environmental monitoring.

## Abstract (ru)

Обзор посвящен пикобирнавирусам (ПБВ) – мелким безоболочечным изометрическим виру- сам, таксономически относящимся к роду Picobirnavirus (PBV) семейства Picobirnaviridae, с геномом, пред- ставленным двумя сегментами двуцепочечной РНК. На основании публикаций за 1988–2019 гг. представ- лена информация о распространенности пикобирнавирусов в природе, о широком спектре поражаемых хозяев. Раскрыт оппортунистический характер ПБВ инфекции и подчеркивается отсутствие ясной картины в понимании роли ПБВ в качестве этиологического агента диареи, поскольку эти вирусы выявляются и при отсутствии симптомов заболевания. Рассматривается концепция, обосновывающая представление о ПБВ инфекции как о хроническом заболевании, обусловленном длительной персистенцией вируса в организ- ме хозяина. Причины высокой частоты выявления ПБВ у людей и животных объясняются влиянием таких факторов, как стрессовый синдром, физиологическое состояние, иммунный статус и возраст хозяина при первичном инфицировании. Отмечается возможный зоонозный характер ПБВ инфекции человека, природа которого объясняется способностью этих вирусов к межвидовой трансмиссии, приобретенной в ходе эво- люции благодаря реассортации сегментов генома разных вирусов, инфицировавших одного хозяина. При- водятся данные, доказывающие принадлежность ПБВ к вирусам эукариот, а также ставящая эти факты под сомнение гипотеза о возможной принадлежности ПБВ к вирусам бактерий. Подчеркнута необходимость ак- тивизации работ по выявлению ПБВ в связи с их широким распространением, несмотря на сложность из-за отсутствия системы для их культивирования. В качестве основных способов их детекции рассмотрены две стратегии ОТ-ПЦР ПБВ. Приведена характеристика геномов отдельных представителей рода, выделенных от разных хозяев. Акцент сделан на целесообразности разработки праймеров с более широкой специфично- стью для увеличения диапазона выявляемых представителей рода ПБВ в связи с огромным разнообразием их генотипов. Подчеркивается важность эффективного мониторинга распространенности ПБВ для изуче- ния их зоонозного и антропонозного потенциала с помощью метагеномного анализа, а также возможность использования этих вирусов в качестве перспективного маркера для мониторинга за чистотой окружаю- щей среды.

## History of the PBV discovery

In 1988 in Brazil when human fecal samples collected during acute gastroenteritis outbreaks were subjected for detection of segmented rotavirus genomes by polyacrylamide gel electrophoresis (PAGE), two band profiles were revealed (Pereira et al., 1988a). Similar profiles were found when examining intestinal contents of rats (Pereira et al., 1988b). These segments were double stranded RNA (dsRNA). Their length was estimated by electrophoretic mobility at about 2.6 and 1.5 kbp for the slow- and fast-migrating segments, respectively. This RNA was cosedimented in the caesium chloride gradient at a density of 1.39–1.40 g/ml with uniform particles ~35 nm in diameter with an indistinct surface structure, detected by electron microscopic examination of samples. The authors proposed the name “picobirnaviruses” (from pico (‘small’), bi (‘two’), and rna (‘RNA’)) for new, previously undescribed small viruses with a bisegmented RNA genome, in contrast to the known larger birnaviruses that infect birds, fish, insects, and mollusks.

Follow-up studies showed a widespread prevalence of picobirnaviruses (PBVs) that were found in the feces of terrestrial and marine mammals, reptiles, birds (Ganesh et al., 2014; Malik et al., 2014; Conceicao-Neto et al., 2016; Navarro et al., 2018), in the respiratory tract of pigs (Smits et al., 2011) and humans (Smits et al., 2012), in fish, invertebrates (Delmas et al., 2019), fungi (Yinda et al., 2018), and, according to recent data, in bacteria (Krishnamurthy, Wang, 2018). The chronology of PBV detection in humans and animals according to data from 1988 to 2018, inclusive, is presented in the Table.

**Table 1.**
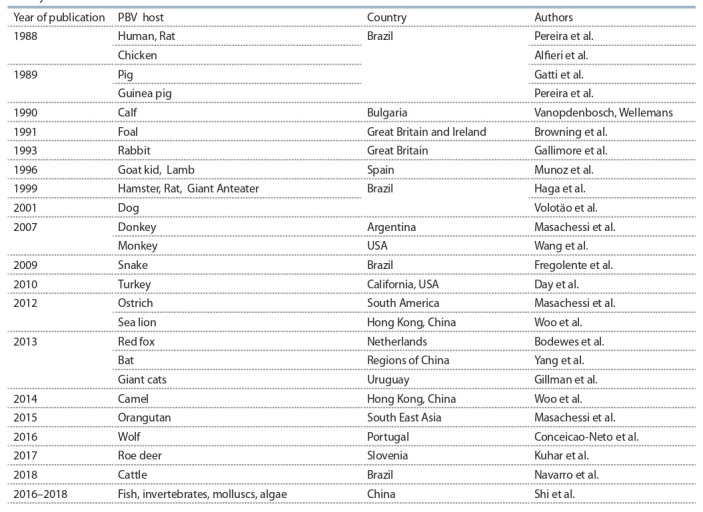
History of the detection of PBV

## Taxonomy

Picobirnaviruses (family Picobirnaviridae) is a family of non-enveloped small spherical viruses, according to Baltimore’s classification, belonging to the class III of viruses with a double-stranded RNA genome (https://viralzone.expasy.org). This new viral family is composed of only one viral genus, Picobirnavirus, uniting the viruses of five genetically variable clusters (genogroups) (Luo et al., 2018)

The two species under the genus are Human Picobirnavirus and Rabbit Picobirnavirus, where the former one is nominated as a type species and the latter one as designated species by the International Committee on Taxonomy of Viruses (ICTV) in 2008 (Delmas et al., 2019). Picobirnaviruses from other hosts have not yet been approved as type species and are considered unclassified (Malik et al., 2014; Takiuchi et al., 2016). In addition, it is not completely clear whether eukaryotes or bacteria are the natural hosts of PBVs (Krishnamurthy, Wang, 2018). Information about the taxonomy of Picobirnaviridae is available in the ICTV report summary by reference at the link www.ictv.global/ report/picobirnaviridae. Taxonomically, the closest relatives of PBVs are viruses of the family Partitiviridae, that have a similar capsid structure and genome organization (Delmas et al., 2019). Natural hosts of partitiviruses are fungi and plants (Vainio et al., 2018).

## Structural and molecular organization of PBV

**Fig. 1.**
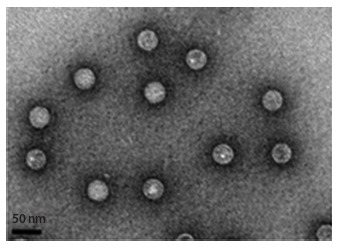
Electron microscopic images of purified and concentrated particles of PBV (Collier et al., 2016). The hexagonal contour of the particles proves their icosahedral symmetry.

**Fig. 2.**
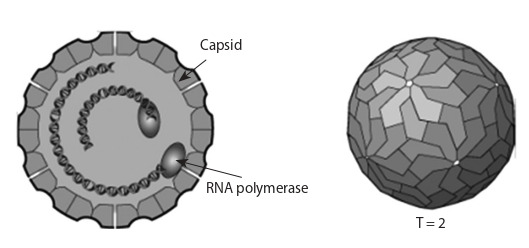
Virion PBV (https://viralzone.expasy.org/by_species/740).

Morphologically PBV virions are small non-enveloped particles 35–40 nm in diameter with indistinct surface structure (Fig. 1) (Rosen et al., 2000; Wakuda et al., 2005; Duquerroy et al., 2009; Collier et al., 2016). A capsid has a cubic (icosahedral) type of symmetry, has a 30-sided (triacontahedral) organization, and consists of 60 asymmetric subunits that are homodimers (Fig. 2). These subunits form 60 protrusions on the surface of the capsid. Since each of the subunits is a dimer, in total, the capsid consists of 120 protein molecules, which makes it possible to attribute PBVs to structures with a triangulation number “T = 2” when characterizing the virion symmetry. In the capsid there are channels connecting the internal cavity with the virion surface (Duquerroy et al., 2009).

Significantly different in capsid architecture from higher eukaryotic viruses with dsRNA (Reoviridae), PBVs are similar to dsRNA viruses of the family Partitiviridae (Ochoa et al., 2008). However, according to recent data, in contrast to partitiviruses, PBVs can infect prokaryotic cells in addition to fungal host cells (Knox et al., 2018).

**Fig. 3.**
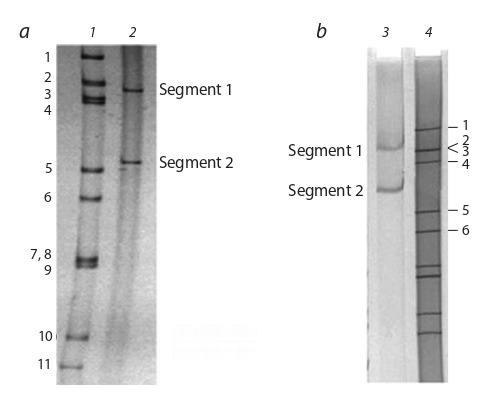
“Large” (a) and “small” (b) migration profiles of PBV RNA segments in PAGE compared to the rotavirus RNA migration profile (Wakuda et al., 2005; Ghosh et al., 2009). 1 – monkey rotavirus SA-11; 2 – human PBV Hy005102; 3 – PBV cattle RUBV-P; 4 – human rotavirus DS-1.

PBV genome consists of two dsRNA segments whose sizes differ in viruses isolated from different animal species. In polyacrylamide gel electrophoresis (PAGE) these segments diverge relative to each other at a certain distance. In this case, two types of electrophoregrams are formed: with a larger (segments higher) genome profile (the segments 1 and 2 correspond to 2.7 kbp and 1.9 kbp, respectively) and with a shorter genome profile (segments lower, 2.2 kbp and 1.2 kbp) (Fig. 3) (Duquerroy et al., 2009).

The larger segment 1 of the PBV genome can consist of two or three open reading frames (ORF). It should be noted that in most studies, the segment 1 of the PBV genome is schematically represented as two ORFs, for example, in the PBV genome schemes of human (Rosen et al., 2000), pig (Carruyo et al., 2008), bull (Ghosh et al., 2009), sea lion (Woo et al., 2012), fox (Bodewes et al., 2013), turkey (Verma et al., 2015), horse (Li et al., 2015), gorilla (Duraisamy et al., 2018), marmot (Luo et al., 2018). In a number of other works, the segment 1 consists of three ORFs, for example, in the genome schemes of rabbit, roe deer and chicken in studies of Green et al. (1999), Kuhar et al. (2017) and Boros et al. (2018), respectively.

**Fig. 4.**
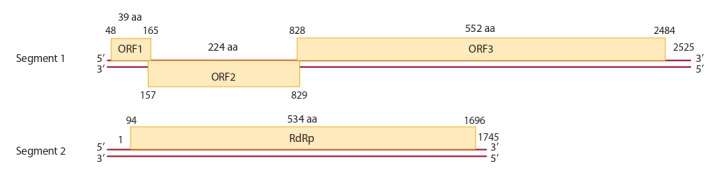
Schematic representation of the location of genes in segments 1 and 2 of dsRNA strain Hy005102 of human picobirnavirus (King et al., 2012). Numbers indicate nucleotide positions; aa – aminoacid.

In schemes where the segment 1 consists of three ORFs, the smallest ORF1 encodes a polypeptide comprising only a few tens of amino acids. For example, in the scheme of the human PBV genome (strain Hy005102) presented by King et al. (2012), ORF1, preceding two larger frames ORF2 and ORF3 encoding peptides of 224 and 552 amino acids, consists of only 39 codons (Fig. 4). In the PBV genome schemes of rabbit, roe deer and chicken in the studies of Green et al. (1999), Kuhar et al. (2017) and Boros et al. (2018) ORF1 is slightly larger and comprises 55, 63 and 188 codons, respectively. The functionality of ORF1 is unclear, and therefore its presence is not always mentioned by other researchers (Boros et al., 2018).

ORF2 in schemes with the segment 1, consisting of three ORFs, encodes the so-called hydrophilic peptide containing conserved repeating sequences, which is one of the main features of the PBV genome (Boros et al., 2018). There is an assumption that two frame shifts occur during translation to generate one long protein (Green et al., 1999).

The third, the largest reading frame (ORF3) in the PBV segment 1 encodes a virus capsid protein of 552–591 amino acids. Frames may overlap. In the scheme of King et al. (2012), the three ORFs overlap at eight (ORF1–ORF2 junction) and one (ORF2–ORF3 junction) nucleotides.

The shorter segment 2 of the PBV genome contains one ORF encoding the enzyme RNA-dependent RNA polymerase (RdRp) (see Fig. 4). Depending on differences in RdRp gene specificity in the segment 2 of the PBV genome, PBVs are divided into genogroups (Malik et al., 2014).

In addition to the “typical” PBVs described above, “atypical” PBVs were detected using PAGE, first in the feces of calves (Vanopdenbosch et al., 1989), and later in the human feces (Gallimore et al., 1995а; Khramtsov et al., 1997). “Atypical” PBVs have a smaller genome than “typical” ones (RNA segment sizes range from 1.75 to 1.79 kbp and from 1.37 to 1.55 kbp), and differ in the location of genes encoding functional proteins (Gallimore et al., 1995b; Khramtsov et al., 1997). If in “typical” PBVs, the segment 2 encodes the viral RNA polymerase, and the segment 1 encodes a capsid protein, then in “atypical” ones it is the opposite.

The PBV genome is usually segmented (Duquerroy et al., 2009; Delmas et al., 2019). However, several nonsegmented PBV genomes belonging to different genetic clusters have recently been described, particularly PBV genomes of horses (Li et al., 2015), of Himalayan marmots (Luo et al., 2018), fish and invertebrates (Shi et al., 2016). It was found that there is an evolutionary relationship between PBVs with a segmented and non-segmented genome, which is due to the possibility of transition from one form to another. It turned out that the PBV genome of marmots can be both segmented and non-segmented. A non-segmented genome contains three ORFs – ORF1, ORF2, ORF3 encoding a hydrophilic protein, a capsid protein, and RdRp, respectively (Luo et al., 2018).

Sequencing studies of PBVs have shown that PVBs are extremely variable (Bányai et al., 2003, 2008; Carruyo et al., 2008; Smits et al., 2011; Ganesh et al., 2014; Malik et al., 2014; Li et al., 2015; Duraisamy et al., 2018; Luo et al., 2018). PBV genome variability is explained by characteristic genetic variability, caused not so much by a change in the primary structure as a result of mutations, but by the genome segment reassortment (Woo et al., 2019).

Until 2014, researchers identified two main PBV genogroups based on the study of short incomplete RdRp gene sequences (Malik et al., 2014). When analyzing the PBV sequences presented in the database GenBank (https://www.ncbi.nlm.nih.gov/nuccore), as of 17.07.2014 (Malik et al., 2014) it was noted that 83.11 % of the sequences belong to the PBV genogroup I (GI), and only 2.52 % – to the genogroup II (GII), which corresponds to the prevalence of representatives of these genogroups in nature.

However, further research results have shown that PBVs exhibit a high level of genetic diversity that is not reflected by just two genogroups. In 2014–2015, there were reports of the discovery of a new genetic PBV variant in human stool samples (Smits et al., 2014) and in the environment (Zhang S. et al., 2015). The new human PBV showed low similarity (19.4–26.1 %) in the sequence of amino acids in the RdRp gene product with the human PBV genogroups I and II, which allowed them to be assigned to the genogroup III (PBV GIII/Homo sapiens/VS6600008/2008/NL/ KJ206569 in GenBank). In 2015 picobirnaviruses were identified in horses, which formed separate clusters and were assigned to two genogroups GIV and GV during phylogenetic analysis of 450 aa RdRp protein sequence (Li et al., 2015).

Thus, picobirnaviruses are currently classified into five genogroups GI–GV based on RdRp sequences. At the same time, the intra-genogroup amino acid identities range from 44.8 to 97.1 %, whilst the inter-genogroup amino acid identities range from 21.6 to 30.8 % (Li et al., 2015). In humans, four PBV genogroups – GI, GII, GIII (Smits et al., 2014) and GV (Ng et al., 2014) were identified. All five PBV genogroups were identified in the marmot (Luo et al., 2018). Genogroup GIII PBVs were found in diatoms and invertebrates (Shi et al., 2016, 2018; Delmas et al., 2019).

According to the currently accepted nomenclature of picobirnaviruses proposed by Fregolente et al. (2009), the name of the strain begins with the genogroup name (GI–GV), followed by the abbreviation PBV, the common name of the host species, the three-letter country code, the name of the strain and the year of isolation, separated by a slash. Using this nomenclature, PBV strains detected in humans and turkeys received the following designations – GI/PBV/human/BRA/PBV_RVH275/2013 (human PBV) и GI/PBV/turkey/USA/MN-1/2011 (turkey PBV).

## Opportunistic character of PBV-infection. On PBV persistence

In spite of the fact that PBVs are often detected from humans and animals with diarrhea separately and co-infection with other pathogens (Ganesh et al., 2014), the role of PBVs as causative agents of intestinal disorders has not been established, since these viruses are detected in asymptomatic cases (Masachessi et al., 2007; Martínez et al., 2010; Verma et al., 2015). For example, Verma et al. (2015) showed that the excretion of PBVs in turkeys was not associated with the symptoms of diarrhea. Of the 80 fecal samples from poultry with diarrhea and 40 without diarrhea, 39 (48.8 %) and 23 (57.5 %) were positive for PBVs, respectively.

It is not shown, that PBVs might have etiological relation with diarrhea in humans and animals infected with other pathogens that cause gastroenteritis. The adaption of the virus to grow in the cell culture and experiments on gnotobiotic animals will be essential to establish the etiologic role of PBVs (Ganesh et al., 2014).

Since the end of the 90s of the last century, PBVs have been identified as opportunists for their ability to provoke diarrhea in animals infected with the primary pathogen or humans with weakened immunity and later to manifest themselves as a chronic disease with or without signs of diarrhea (Giordano et al., 1998; González et al., 1998; Martínez et al., 2003; Masachessi et al., 2007, 2012; Ghosh et al., 2009). There is a concept about the opportunistic (conditionally pathogenic) nature of PBV infection, which is presented in the review published by Ganesh et al. (2014) and other reports based on the results of examination of healthy animals for the presence of PBVs (Masachessi et al., 2007; Carruyo et al., 2008; Martínez et al., 2010). According to this concept, the chronic nature of PBV infection is explained by the long-term persistence of the virus in the host body (Ganesh et al., 2014). The persistent nature of PBV infection is manifested by periods of silence, in which the virus is not detected even using highly sensitive methods, intermingled with periods of viral activity. In this case, adult animals infected with PBVs could be asymptomatic PBV carriers or may be persistently infected and serve as reservoirs of the infection. In particular, research results obtained by Carruyo et al. (2008) shown that PBVs can infect piglets 7 to 56 days of age (the PBV prevalence among piglets has been estimated to be around 10 to 12 %).

The virus excretion level by virus carriers is affected by a number of factors such as stress syndrome, physiological status, age of the primary infected individual, immune status of the hosts, and environmental conditions (biotic and abiotic factors). These factors contribute to an increase in the viral load. When taking samples based on these factors, the detection of PBVs by the PAGE was observed with a higher frequency.

The dependence of the PBV excretion level on the stress caused by keeping animals captive was observed in African green monkeys from the Caribbean Islands in 2014–2015 (Gallagher et al., 2017). 270 fecal samples were collected from wild (160 individuals) and captive (110 individuals) monkeys subjected to PAGE for PBVs. 16 samples (14.5 %) from captive monkeys kept under stressful conditions were indicative for PBVs. None of the fecal samples from wild monkeys was tested positive for PBVs (Gallagher et al., 2017).

The dependence of the PBV excretion level on the physiological status of pigs, in particular during the reproduction period, was studied by researchers in Argentina (Martínez et al., 2010). Observations showed that PBVs persisted in the host as a permanent infection, with periods of low and high PBV excretion intermingled with periods of silence. Low PBV excretion levels were detected by reverse transcription followed by polymerase chain reaction (RT-PCR) throughout the study period. In case of sharp increase in the PBV excretion level in pigs, observed during farrowing and lactation periods, the virus was detected not only by OTP-PCR, but also by PAGE. PBV detection rate was highest in the group of sows sampled within the lactogenic period (38.02 % of samples collected from 71 sows), followed by pregnant sows at the final stage of gestation (15.09 % of samples collected from 53 pregnant sows) (Martínez et al., 2010).

The dependence of the PBV excretion level on the age of the animal is shown. From 289 fecal samples of individual calves between 5 and 60 days of age analyzed by PAGE the PBVs were detected in 24 (8.3 %) (Takiuchi et al., 2016.) In the study (Martínez et al., 2010), a high PBV excretion level in pigs was observed in young animals 2–5 months of age (18.42 %), while no excretion was observed in adult male pigs. Studies on turkeys also revealed a change in the frequency of PBV excretion (Verma et al., 2015). 39 of 80 (48.8 %) fecal samples from turkeys were PBV positive. The maximum number of samples was positive in turkeys at 2 weeks of age (20 of 20) followed by 3 weeks of age (15 of 20). A sharp decrease in the number of positive samples (2/20 were positive at 8 weeks of age) indicated a decisive influence of age on this process.

The PBV excretion level is also affected by the host’s immune status. It was found that in people with weakened immunity, PBVs are detected more often. For example, Giordano et al. (1998) when investigating 197 stool samples collected from HIV-infected and noninfected patients with and without diarrhea for the presence of PBVs by PAGE detected PBVs in 8.8 % of 57 HIV-infected patients with diarrhea, but detected it in neither those without diarrhea nor in the group of subjects uninfected with HIV. Further research by these authors reinforced their view of the relationship between PBV excretion levels and diarrhoea in HIV-infected individuals. In a study of 244 stool samples from HIV-infected and uninfected patients with and without diarrhea, PBVs were detected in 14.63 % of 82 HIV-infected patients with diarrhea and it was detected neither in those without diarrhea nor in the group of subjects uninfected with HIV (Giordano et al., 1999). At a certain PBV excretion level, PBVs are detected in HIV-infected patients without signs of diarrhea, for example, González et al. (1998) detected PBVs in 2.3 % of 125 HIV-infected patients without diarrhea (González et al., 1998).

The PBV excretion level may be influenced by biotic environmental factors such as primary pathogens. In some studies, for example, PBVs are most often isolated as coinfected agents with a number of diarrheal causes, such as rotaviruses (Kuhar et al., 2017), or noroviruses (Bányai et al., 2003). These studies indicated that PBVs might have played synergistic effect in association with the primary enteric causes (Malik et al., 2014; Kylla et al., 2019).

Climate factors such as sunlight (Masachessi et al., 2015), temperature, and humidity (Ribeiro et al., 2014) also affect the viral activity. These factors that affect the PBV excretion level should be taken into account when identifying them.

## Zoonotic nature of PBV-infection. Capacity for interspecies transmission

Most human viral diseases are of zoonotic origin. The zoonotic character of the PBV infection is indicated by the detection of genetically related PBVs in humans and animals. In particular, from pigs in Hungary, Venezuela and Argentina genogroup I PBVs were detected which, when sequencing the genome, showed genetic similarity to the human genogroup I PBVs (Bányai et al., 2008; Carruyo et al., 2008; Giordano et al., 2011). There have been reports of PBV strains found in children in Calcutta that are genetically related to porcine PBV strains (Ganesh et al., 2010, 2011a). Equine PBV strains isolated from the faeces of foals in Calcutta (India) showed a genetic relationship with human strains from the same city (Ganesh et al., 2011b). The detection of genetically similar PBVs in humans and foxes (Lojkić et al., 2016), in humans and bats has been reported. For example, the report (Yinda et al., 2019) noted that the cause of zoonotic transmission of PBVs to humans from bats in Cameroon is the hunting and eating of bats (Yinda et al., 2019).

Zoonotic PBV transmission is one of the variants of interspecies transmission. The capacity for interspecies transmission was acquired by PBVs in the course of evolution due to the reassortment of segments of their genome while simultaneously infecting a single cell by PBVs of different species (McDonald et al., 2016). The genetic lability caused by reassortment could lead in the course of evolution either to genetic convergence of PBV strains belonging to different hosts or, conversely, to genetic divergence – genetic distance of PBV belonging to hosts of the same species (Lojkić et al., 2016).

The capacity for interspecies PBV transmission is confirmed both in the cases of zoonotic infections, and in cases of infection of animals with human PBVs, for example, young pigs (Carruyo et al., 2008) or horses (Ganesh et al., 2011b). The genomes of some porcine PBV strains were found to be identical to those of human genogroup I PBVs (Ganesh et al., 2014). In 2011, genetically similar PBVs were found in the respiratory tracts of pigs and humans (Smits et al., 2011, 2012).

Genetic divergence of related PBV strains was demonstrated by Zhang B. et al. (2014). Thus, three of four porcine PBVs identified in the study were genetically closer to human PBVs than to previously revealed porcine PBVs.

It is possible to transfer PBV strains from one host to another through fecal-contaminated raw sewage (Symonds et al., 2009). In an environmental study Symonds et al. (2009) showed that picobirnaviruses are potentially useful viral indicators of fecal pollution of naturally impounded bodies since they were found in 100 % of raw sewage samples and 33 % of final effluent samples.

## Hypothesis about the phage nature of PBVM

PBVs commonly found in animal fecal samples are currently thought to be animal viruses, but no animal model or cell culture for PBV propagation has yet been found. Recently, some Indian scientists hypothesized that PBVs are prokaryotic RNA viruses (Krishnamurthy, Wang, 2018). The hypothesis is based on the fact, that like prokaryotic viruses with the RNA-genome, in the PBV genome, there are conserved ribosomal binding site (RBS) sequences called Shine–Dalgarno sequences upstream of the three presumed ORFs of the segment 1 and a single presumed ORF of the segment 2. Such sites are 6-mers (AGGAGG) preceding codons that initiate the translation of the viral genome sequences. In bacterial viruses, these 6-mers are ribosomal binding sites and serve to enhance the translation efficiency of viral proteins. For example, such sites are present in the genome of some bacteriophages of the family Cystoviridae with a segmented dsRNA genome (Boros et al., 2018).

Findings obtained by Adriaenssens et al. (2018) support the hypothesis that PBVs are bacteriophages. The authors demonstrated a high incidence of 6-mer motif AGGAGG in the PBV genome. In contrast, the different families of eukaryotic viruses analyzed in that study only showed a low incidence of SD-sequences, which were mostly 4-mers (АGGА, GGAG, GAGG). Findings supporting the bacteriophage-nature of PBVs were also obtained by Boros et al. (2018), who revealed in the chicken PBV genome the presence of conserved prokaryotic Shine–Dalgarnolike (SD-like) sequences upstream of the three presumed ORFs of the segment 1 and a single presumed ORF of the segment 2.

If we assume that PBVs are prokaryotic viruses, we can explain their widespread prevalence and a broad host range. Bacteriophages are widely distributed in nature. They are found in water, soil, food products, various excretions of humans and animals, that is, where bacteria are found.

The identification of PBV strains with genetically related genome sequences in different animal species can be explained by assuming that PBVs might actually infect bacteria that populate the enteric tract of vertebrates and invertebrates. At the same time, the authors of the phage hypothesis (Krishnamurthy, Wang, 2018) believe that PBVs replicate in bacteria of a certain type, in the genome of which there is a prokaryotic SD-sequence in most genes (more than 10 %). Such bacteria are Firmicutes in the genome of which there are more than 80 % of genes with prokaryotic SD-sequence (Omotajo et al., 2015).

The authors point out, that, even viral families that include species whose genomes are enriched for SDsequences, are exclusively prokaryotic viral families. The acquisition of immunity against PBVs by infected animals also does not contradict the phage hypothesis, since it is established that immune responses can be raised against bacterial viruses (Dabrowska et al., 2005; Górski et al., 2006). It is possible that PBVs cause an immune response to infection not of human cells, but of the bacterial cells that make up its microbiome, which again does not exclude the possibility that PBV are prokaryotic viruses.

The possession of a capsid protein with perforation activity, which means the ability to translocate through the cell membrane (Duquerroy et al., 2009), as proof that it can infect animal cells, also does not contradict the phage hypothesis, since it is known that representatives of the bacterial RNA-virus family also have the ability to exit the cell (Reed et al., 2013). The process of interaction of human and animal PBVs with the host cell is similar to the process of interaction of a virulent phage with a bacterium, which proceeds in several stages – penetration into the bacterial cell, autonomous reproduction in it and lysis of the bacterium. Consequently, the perforation of liposomes by PBVs does not exclude that they may be prokaryotic RNA-viruses.

Summing up the arguments in favor of the phage hypothesis, we can conclude that, perhaps, PBVs belong to a new family of RNA-viruses that infect a certain type of bacteria with a genome with a high content of SD-sequences (more than 80 %). These bacteria populate the intestinal tract of animals and humans and they can be bacteria Firmicutes, containing most genes with SD-sequences.

These arguments strongly suggest that PBVs can actually infect prokaryotes, not eukaryotes. And if PBVs actually infect bacteria, then it is necessary to change the approach to their study – to direct efforts towards finding a host for their replication among prokaryotic cells, rather than eukaryotic ones. Separation prokaryotic and eukaryotic virus families by the frequency of the presence of SD-sequences in the genome allows identifying new prokaryotic virus families.

Thus, the final proof of the phage nature of PBVs requires the selection of host cells for its replication. In all likelihood, until successful cultivation of PBVs in specific bacterial cultures is achieved, the phage nature of PBVs remains hypothetical. Given the fact that gut microbiome consists of several hundreds of mostly uncultivable bacteria the identification of true bacterial or archeal host(s) of PBVs (if any) will be challenging (Boros et al., 2018).

## RT-PCR-amplification strategies for PBV sequencing and genotyping

Before the use of nucleic acid amplification methods, the detection rate of PBVs remained extremely low, because of low sensitivity of the method of genomic RNA PAGE (Masachessi et al., 2007). In addition, PBVs are very labile agents. It is shown that samples tested PBV positive by PAGE, become negative after several freezing–thawing procedures (Gallimore et al., 1995a).

The low frequency of detection of PBVs by PAGE is evidenced by the work of Argentine virologists (Giordano et al., 2008). These authors collected 2224 stool samples from children with diarrhea over a 25-year period from January 1977 to December 2002. Only two samples (0.09 %) were tested PBV positive by PAGE. Similar results were obtained in our studies on the detection of rotaviruses by PAGE, in stool samples from children under 14 years of age with acute intestinal infection, who were admitted to infectious hospitals in the city of Nizhny Novgorod, Nizhny Novgorod region. For the period 1994–2001 PBVs were found in only 3 of 4535 samples tested (Novikova et al., 2003). Subsequently, from July 2006 to January 2010, PBVs were detected in 0.08 % of 3645 stool samples from children with gastroenteritis (Epifanova et al., 2010).

The use of PAGE limited the frequency of PBV detection, since at the low viral load virus excretion was undetectable in most clinical samples (Gallimore et al., 1995a; Giordano et al., 1998). A low frequency of PBV detection by PAGE was reported by Cascio et al. (1996), Pereira et al. (1993) and Ludert, Liprandi (1993), in a study of sporadic gastroenteritis cases in children in Italy (0.43 %), Brazil (0.5 %) and Venezuela (0.5 %), respectively. However, in outbreaks of gastroenteritis, when the virus excretion level was high, the virus was detected by PAGE with a significantly higher frequency. For example, Pereira et al. (1988a) reported a frequency of human PBV detection in outbreaks of gastroenteritis in Brazil up to 20 %.

The introduction of RT-PCR amplification and sequencing technologies has contributed to an increase in the PBV detection rate in various wildlife objects. For example, a total 60 % (87/144) of the fecal samples from newborn piglets tested were found to be PBV positive by RT-PCR (versus 27 % by PAGE) (Carruyo et al., 2008). The information presented in the paper on the prevalence of porcine PBVs in Argentina (Martínez et al., 2010) also demonstrates the significantly greater capabilities of the RT-PCR method compared to PAGE. If the PAGE method in this study detected PBVs only at high PBV excretion level conditioned by age (primary infection), and host physiological status, low PBV excretion levels were detected by RT-PCR throughout the entire study period.

The application of these methods allowed to establish that PBVs are more widespread in nature than it was previously discovered (Boros et al., 2018). In particular, a group of researchers from the Netherlands when testing 83 stool samples from patients with diarrhea by RT-PCR confirmed 17 samples positive for genogroup I PBV sequences (20 %) (van Leeuwen et al., 2010). By the same method, a high proportion of PCR positive samples (23.4 %) was detected in a total of 77 bovine fecal samples from different Brazilian regions analyzed (Navarro et al., 2018). The high frequency of infection in the sheep flock in Brazil evaluated by RT- PCR was established where 62 % of the analyzed fecal samples were PBV-positive (Kunz et al., 2018).

Most researchers use two specific amplification strategies to detect PBVs: the single and double primer PCR amplification strategies. The first amplification strategy is based on the ligation of a viral RNA as a matrix with an oligonucleotide as an adapter, followed by the synthesis of cDNA on this matrix using a complementary adapter primer. This method developed by Lambden et al. (1992) to carry out amplification of the viruses with segmented dsRNA genomes was further used by researchers for the amplification of the PBV genome (Wakuda et al., 2005; Ghosh et al., 2009; Wang et al., 2012; Boros et al., 2018). In particular, Wakuda et al. (2005) applied the modified single primer strategy to prepare full-length cDNAs corresponding to RNA segments 1 and 2 of a human picobirnavirus (strain Hy005102).

The single primer PCR amplification strategy is usually applied to characterize full-length PBV genome segments. For PBV genotyping based on short genome specific fragments to characterize the strain with the definition of its genogroup, the second strategy of specific amplification is used, which involves the use of a pair of primers flanking the selected viral genome fragment. Reverse transcription- PCR detection assays were developed with primers targeted to the genome segment of 2 PBV strains – 4-GA-9 and 1-CHN-97 isolated in the United States and China, respectively (Rosen et al., 2000).

In the case of human PBVs, the primers are targeted to specific conserved sites (motifs) in the PBV genome segment 2 encoding RNA-dependent RNA polymerase. For detection of the genome group I, direct and reverse primers PicoB25 and PicoB43 are used, which flank the 201 bp (in positions 665–679 and 850–865 of the segment 2) fragment of the RdRp gene. To detect the genome group II, a pair of primers PicoB23 and PicoB24 flanking the 369 bp (in positions 685–699 and 1039–1053) fragment of the RdRp gene is used.

These primers can be used not only for human PBV genotyping (Rosen et al., 2000), but also for the genetic characterization of PBVs in some animals, in particular pigs (Bányai et al., 2008). However, they are not able to recognize the full range of PBV strains circulating among humans, pigs, and other hosts due to their narrow specificity (Bányai et al., 2008; Carruyo et al., 2008; Ganesh et al., 2010, 2011a; Martínez et al., 2010). In this regard, primers flanking other degenerated parts of the RdRp gene (Carruyo et al., 2008; van Leeuwen еt al., 2010; Verma et al., 2015; Wilburn et al., 2016; Woo et al., 2019), as well as primers with a wider range of detection of representatives of the genus Picobirnavirus, were later developed to expand the specificity in detecting sequences of segments of the PBV genome (Malik et al., 2017; Ghosh et al., 2018; Kleymann et al., 2020).

In particular, a RNA polymerase gene based RT-PCR diagnostic assay was developed for detecting PBVs in early stages of infection in a wide range of hosts including animals and humans (Malik et al., 2017). Through RdRp gene nucleotide sequences alignment analysis, the conserved regions of PBV were used for generating primers universal for the genus. The best results for RT-PCR specificity and sensitivity were given by a primer set with sense primer PBV-7F (position 754–771 of the segment 2), and antisense primer PBV-7R (position 1011–1028) flanking the 275 bp amplicon. The developed assay made it possible to effectively amplify this fragment of the RdRp gene in all tested PBVs infecting different host species, and did not give false positive results when tested on other viruses.

The use of primers with broad specificity, in addition to expanding the range of detection of representatives of the genus Picobirnavirus, allows amplifying sequences of greater length. In particular, Ghosh et al. (2018) using a wide-specific terminal primer, amplified a region of the RdRp gene overlapping several conserved sites and as a result were able to characterize the complete genome segment 2 of rat PBVs, providing important information on genetic diversity and evolution of PBVs in rats.

Recently, Kleymann et al. (2020) to detect PBVs from different mongoose species, have designed a pair of widely specific primers PBV 1.2FP and PBV 1.2RP that amplify a significant part of the RdRp gene (1229 bp from ~1700 bp). This pair of primers allows detecting PBVs isolated from different hosts and having differences in primary structure (Kleymann et al., 2020). To identify genovariants belonging to the genogroup I among positive samples of mongoose PBVs the authors used a combination of the known reverse primer PicoB43 (Rosen et al., 2000) and the forward primer PBV-7F, which allowed amplifying the 390 bp fragment of the segment 2.

In recent years, metagenomic analysis has become widely used in the diagnosis of viral infections (Adriaenssens et al., 2018; Boros et al., 2018; Duraisamy et al., 2018; Yinda et al., 2019; Wille et al., 2019). The method of metagenomic analysis is based on next-generation sequencing (NGS) technology that allows identifying all the genetic material present in an environmental sample, consisting of the genomes of many individual organisms – metagenome. In contrast to PCR technologies that require reference sequences that can only detect known viruses, the metagenomic analysis method can detect viruses with a new genotype (Adriaenssens et al., 2018). Moreover, new genotypes can be detected more often than known ones (Duraisamy et al., 2018; Wille et al., 2019).

The metagenomic analysis characterizes both the viral diversity and the frequency of occurrence of individual metagenome viruses. For example, according to Adriaenssens et al. (2018), the raw sewage metagenome contained representatives of the genera Astroviridae, Caliciviridae, Picobirnaviridae, Picornaviridae, with only PBVs being detected in 100 % of raw sewage samples in this study. In the publication Boros et al. (2018) reported that using the method of metagenomic analysis of the total number of read sequences (13016) present in the metagenome, which is a fecal sample of a chicken, in 516 reads were identified PBVs. In a study Yinda et al. (2019) using metagenomic analysis of fecal samples from Cameroonian residents with and without signs of gastroenteritis (after contact with bats), found that up to 28 out of the 63 pools contained reads annotated as Picobirnaviridae with most of the positive pools from individuals in age groups above 20. These facts indicate a fairly high level of PBV occurrence in wildlife.

## Conclusion

Summarizing the information presented in the review, obtained from publications on PBVs for the period under study, we can conclude:

- PBVs are characterized by a broad host range and ubiquitous distribution.
- The role of PBVs as causes of gastroenteritis is still not fully understood due to the lack of cell culture or animal models for their cultivation. This significantly impedes virus isolation and clinical and pathological studies.
- The frequency of PBV detection varies in different studies, but it is found that it is associated with the physiological status and environmental conditions.
- There is a hypothetical explanation for the spread of PBV infection based on the idea of PBVs as conditionally pathogenic viruses, according to which adult infected hosts with normal immune status can be PBV carriers and serve as reservoirs of viruses without symptoms of diarrhea.
- The detection of PBV strains with genetically related genome sequences in different animals indicates the possible zoonotic nature of the infection for humans and the ability of PBVs to transmit effectively.
- Being the most common in raw sewage, PBVs correlate better than other viruses with the presence of pathogenic viruses dangerous for humans in water bodies and are potentially useful viral indicators of fecal pollution of these water bodies.
- PBVs are significantly different genetically. To date, 5 PBV genogroups have been identified (GI–GV).
- It has been suggested that PBVs can infect prokaryotes, being not mammalian viruses, but a new family of RNA bacteriophages. In support of this assumption, the authors provide convincing arguments showing that PBVs can actually infect prokaryotes, and not eukaryotes, in particular, bacteria Firmicutes. However, until a host is found for PBV propagation, this assumption remains hypothetical.

The presented information allows characterizing PBVs as viruses that are genetically variable with a broad host range, quickly evolving and easily spreading. However, for a more complete study of PBV biology, etiological role in the occurrence of diseases, and pathogenic potential, experiments on gnotobiotic animals are required. Molecular characterization of new PBV strains from different hosts will provide valuable information about the origin, transmission, distribution, and genetic diversity of these quickly evolving dsRNA viruses to study their zoonotic and anthroponic potential, and to use as a potentially promising marker for environmental monitoring.

## Conflict of interest

The authors declare no conflict of interest.
